# Correlative analysis of lung CT findings in patients with Birt–Hogg–Dubé Syndrome and the occurrence of spontaneous pneumothorax: a preliminary study

**DOI:** 10.1186/s12880-022-00743-3

**Published:** 2022-02-07

**Authors:** Jinjing Yang, Xiaowen Hu, Junjun Li, Guofeng Zhang, Yaqiong Ge, Wei Wei

**Affiliations:** 1 Department of Radiology, The First Affiliated Hospital of USTC, Division of Life Sciences and Medicine, University of Science and Technology of China, Hefei, 230001 Anhui China; 2 Department of Respiratory, The First Affiliated Hospital of USTC, Division of Life Sciences and Medicine, University of Science and Technology of China, Hefei, 230001 Anhui China; 3 GE Healthcare China, Pudong New Town, No.1, Huatuo Road, Shanghai, 210000 China

**Keywords:** Computed tomography, Birt-Hogg-Dubé syndrome, Pneumothorax, Lung cysts

## Abstract

**Background:**

The diagnosis of patients with Birt–Hogg–Dubé (BHD) syndrome is always delayed (even for more than 10 years). Improving the understanding and diagnosis of this disease is vital for clinicians and radiologists. In this study we presented the chest computed tomography (CT) findings of BHD syndrome and offered suggestions for BHD cases with spontaneous pneumothorax.

**Methods:**

Twenty-six BHD patients from 11 families (10 men, 16 women; mean age: 46 ± 12 years, 20–68 years) were included. The clinical features of the patients included pneumothorax, renal lesions, and skin lesions. Twenty-three patients underwent chest CT imaging. The cyst condition of each patient derived from reconstructed chest CT imaging was recorded, including the cyst number, size, volume, pattern, and distribution.

**Results:**

Pneumothorax occurred in 54% (14/26) of patients. Among them, 43% (6/14) had pneumothorax more than twice. However, typical skin and renal lesions were absent. Four patients had renal hamartoma. CT showed that 23 (100%) patients had lung cysts. Pulmonary cysts were bilateral and multiple, round, irregular, or willow-like. And 93.6% of the large cysts (long-axis diameter ≥ 20 mm) were under the pleura, and near the mediastinum and spine. The long-axis diameter, short-axis diameter and volume of the largest cysts were associated with the occurrence of pneumothorax (all P < 0.05).

**Conclusions:**

Chest CT imaging can reveal some characteristic features of BHD syndrome. The occurrence of pneumothorax in BHD patients is closely related to their pulmonary cystic lesions.

## Background

Spontaneous pneumothorax is a clinical emergency often characterized by chest tightness and dyspnea. Traditionally, spontaneous pneumothorax is divided into primary and secondary spontaneous pneumothorax. Primary spontaneous pneumothorax refers to the case without a prior known lung disease [1]. Patients with primary spontaneous pneumothorax are often slender and have a low body mass index. Secondary spontaneous pneumothorax may occur in many pulmonary diseases, such as tuberculosis, cystic fibrosis, primary lymphangioleiomyomatosis, Langerhans cell histiocytosis, and Birt–Hogg–Dubé (BHD) syndrome. Among these, chronic obstructive pulmonary disease and cystic fibrosis are more common. In clinical work, patients often suffer recurrent pneumothorax, and surgeons tend to make more efforts in treating patients and pay less attention to the underlying causes of the disease.

This study aimed to investigate the characteristics of lung lesions of BHD patients and the relationship between the occurrence of pneumothorax and lung cyst features through chest computed tomography (CT) images in these patients. We propose a chest CT examination, not just a chest X-ray, should be conducted for BHD patients and the investigation needs to be extended to family members.

## Methods

### Enrollment of patients

A total of 26 BHD patients from 11 families were selected by BHD screening (including chest CT, gene detection, or a dermatological examination). The clinical data of these patients were collected, including lung, skin, and kidney lesions, pneumothorax, and smoking history.

### CT data

Among the 26 patients, 23 underwent a chest CT scan (Neu Viz 128) without the contrast agent. The scanning parameters were as follows: tube voltage, 120 kVp; tube current, 70 mA; slice thickness, 1.25 mm; FOV 400× 400 mm. CT images were reviewed by two thoracic radiologists (J.Y and J.L with 8 and 6 years of experience in thoracic imaging, respectively) with consensus reached. The chest CT images of these 23 patients were retrospectively analyzed, including bilateral pulmonary cyst distribution, cyst shape, the diameter and volume of the largest cyst, the total number of cysts in different size categories (long-axis diameter ≤ 10 mm, 10–20 mm, and ≥ 20 mm), the number of subpleural cysts in these size categories, and the volume of cysts with a long-axis diameter ≥ 20 mm. The term “subpleural” referred to the area within 10 mm from the mediastinum, chest wall, and interlobar fissure. Assuming that the cyst was a long oval shape, the cyst volume was calculated using the following formula: volume = 4/3*πab*^2^ (a: half of the longest diameter, b: half of the length of the shorter diameter) [2]. Intraclass correlation efficient (ICC) was used to evaluate the consistency of the analystic results by the two physicians, and the results with ICC ≥ 0.8 were used for further analysis.

R statistical software (v. 3.5.1; https://www.Rproject.org) was used for analysis. The Spearman analysis method was applied to examine the correlation between imaging characteristics of pulmonary cysts and the occurrence of pneumothorax.

## Results

### Clinical data

Genetic tests were performed on 24 patients and folliculin (FLCN) mutations were confirmed. Another 2 patients did not undergo genetic tests, but their first-degree relatives were confirmed to have gene mutations; chest CT examination revealed multiple cystic lesions in both lungs of these two patients, which met the diagnostic criteria of BHD, and the diagnosis of BHD was made. The 26 patients (10 men and 16 women) were aged 20–68 years, with a mean age of 46 ± 12 years. Pneumothorax occurred in 54% (14/26) of the patients, among whom 43% had pneumothorax twice or more times, with the highest number of four times. The youngest onset age of pneumothorax was 19 years old. A total of 79% (11/14) of patients with a history of pneumothorax were non-smokers. Four patients had complicated lung tumors (including lung cancer and alveolar cell tumor). None of the patients had typical skin or kidney lesions. Renal hamartoma occurred in four cases and skin epidermoid cyst in two cases.

### CT data

**Fig. 1:**
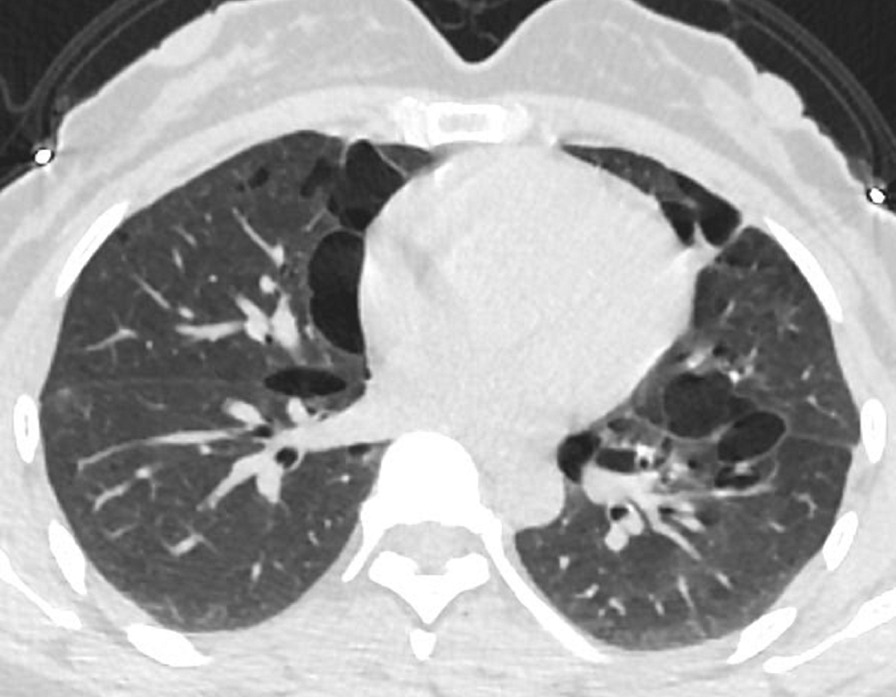
Chest CT images of patients diagnosed with BHD syndrome. Multiple well-circumscribed, thin-walled lung cysts of willow-like, oval, and irregular shapes and varying sizes can be seen. The lung cysts are in the mediastinal subpleural and interlobular fissure areas in both lungs and grow near the mediastinum. Figures 1 and 2 are images of the same patient before (Fig. 1) and during (Fig. 2) pneumothorax

**Fig. 2:**
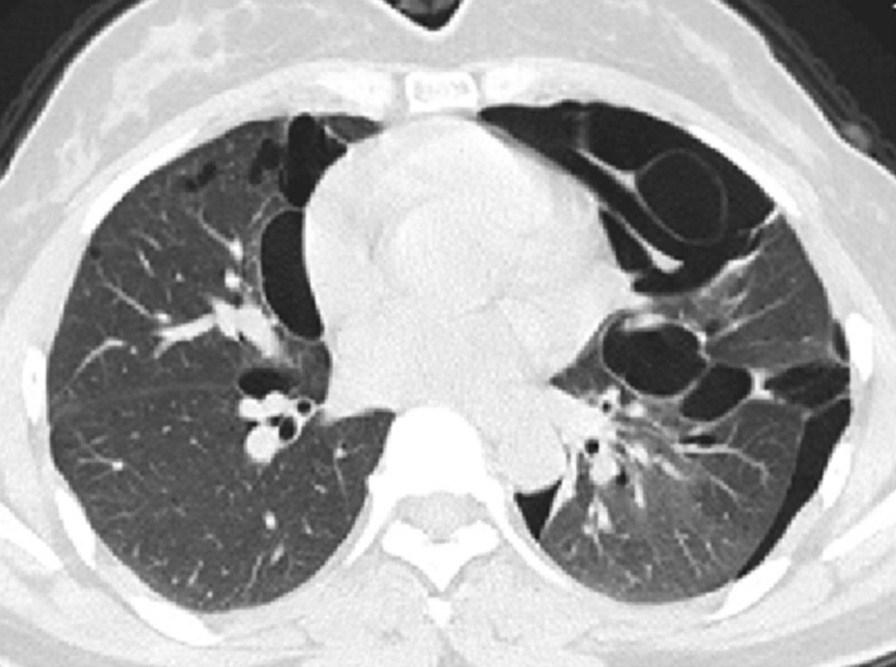
Chest CT images of patients diagnosed with BHD syndrome. Multiple well-circumscribed, thin-walled lung cysts of willow-like, oval, and irregular shapes and varying sizes can be seen. The lung cysts are in the mediastinal subpleural and interlobular fissure areas in both lungs and grow near the mediastinum . Figures 1 and 2 are images of the same patient before (Fig. 1) and during (Fig. 2) pneumothorax

**Fig. 3:**
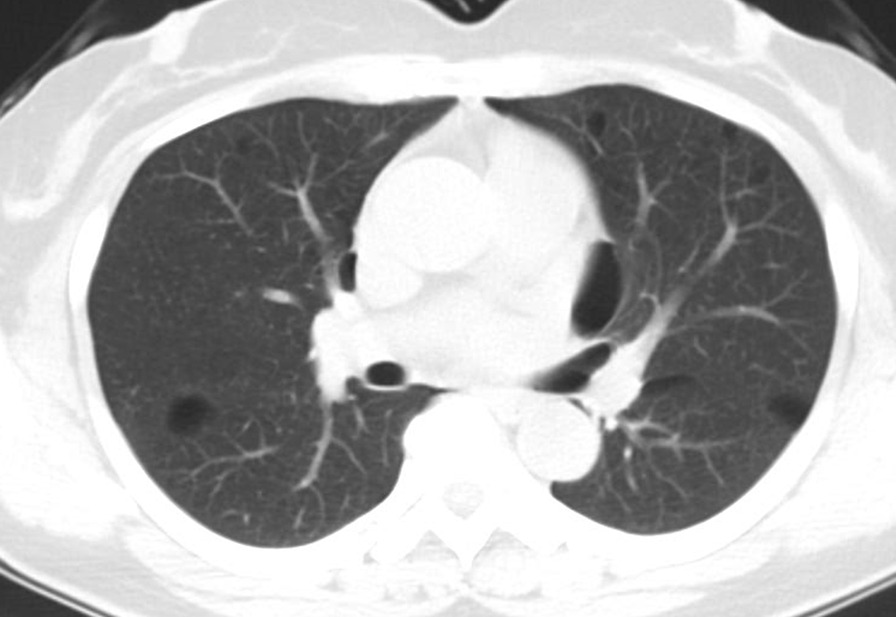
Chest CT images of patients diagnosed with BHD syndrome. Multiple lung cysts of willow-like, oval, and irregular shapes and varying sizes can be seen. The lung cysts are in the mediastinal subpleural and interlobular fissure areas in both lungs and grow near the mediastinum

**Fig. 4:**
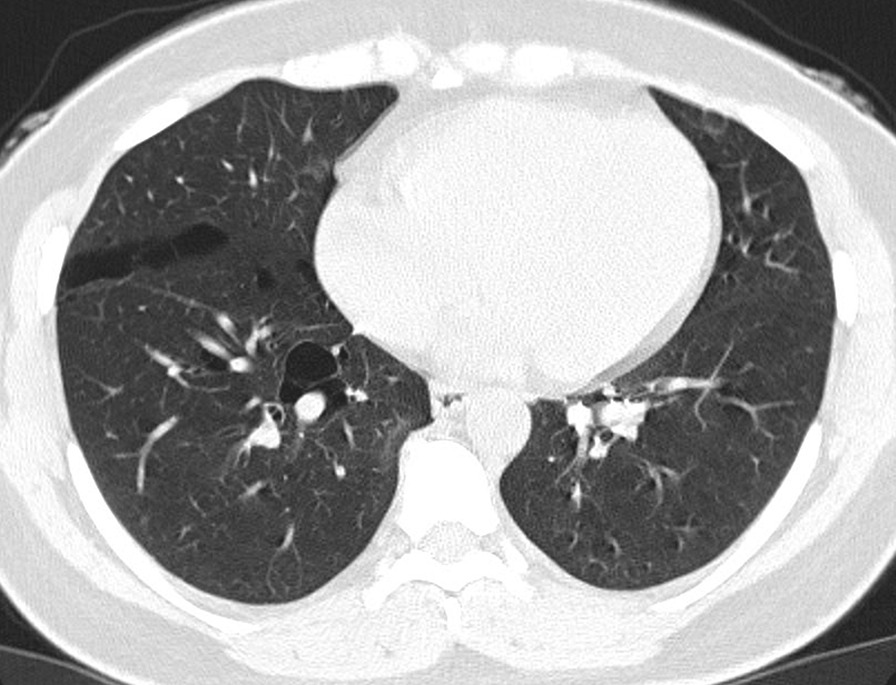
Chest CT images of patients diagnosed with BHD syndrome. Multiple lung cysts ofvarying sizes can be seen. The lung cysts are in the interlobular fissure areas

The final study population included 23 subjects with a total of 2323 lung cysts documented by chest CT examination. Bilateral and multiple pulmonary cysts were found in these patients. The size of the cysts varied, with the diameter ranging from 4 to 110 mm. The cysts were mostly of irregular, willow leaf-like, round, or oval shapes (Fig. 1). Some larger cysts near the mediastinal spine showed plastic changes along the mediastinum and spine. Most cysts were under the pleura of both lungs (Figs. 1, 2, 3), subpleural sacs accounting for 76.3% (1772/2323) of pulmonary sacs. About 93.6% (161/172) of cysts with the long-axis diameter ≥ 20 mm were distributed under the pleura (Table 1), and the mediastinal subpleural ones in accounted for the highest proportion (48.4%; Table 2). Multiple cysts were also observed in the subpleural area of bilateral interlobar fissures (Fig. 4).

**Table 1:** Percentage of mediastinal, chest wall, and interlobar subpleural cysts of different sizes in relation to the size of cysts in both lungs

	Mediastinal subpleural	Chest wall subpleural	Interlobar subpleural
long-axis diameter ≤ 10 mm	23.3% (410/ 1760)	33.5% (590/1760)	16.3% (287/1760)
long-axis diameter 10 mm-20 mm	38.1% (149/ 391)	32.7% (128/391)	12.0% (47/391)
long-axis diameter ≥ 20 mm	45.3% (78 /172)	29.1% (50/172)	19.2% (33/172)

**Table 2:** Percentage of mediastinal, chest wall, and interlobar subpleural cysts of different sizes in relation to the size of cysts of subpleural distribution

	Mediastinal subpleural	Chest wall subpleural	Interlobar subpleural
long-axis diameter ≤ 10 mm	31.9% (410/1287)	45.8% (590/1287)	22.3% (287/1287)
long-axis diameter 10 mm-20 mm	46.0% (149/324)	39.5% (128/324)	14.5% (47/324)
long-axis diameter ≥ 20 mm	48.4% (78/161)	31.1% (50/161)	20.5% (33/161)

For cysts in both lungs, the number of cysts with long-axis diameter ≤ 10 mm accounted for 75.8% (1760/2323), those with long-axis diameter between 10 and 20 mm accounted for 16.8% (391/2323), and those with long-axis diameter ≥ 20 mm accounted for 7.4% (172/2323).

Spearman analysis showed that the long-axis diameter, short-axis diameter, and volume of the largest cysts were significantly associated with the occurrence of pneumothorax (all P < 0.05; Table 3). There were no significant differences in the total number of cysts in both lungs, and the total numbers of cysts in the left, right, and both lungs with the cysts’ long-axis diameter ≤ 10, 10–20, and ≥ 20 mm between the pneumothorax group and non-pneumothorax group. There were no significant differences in the number of subpleural lung cysts with a long-axis diameter ≤ 10, 10–20, and ≥ 20 mm among the mediastinum, chest wall, and interlobar pleura. The total long-axis diameter, short-axis diameter, and volume of the cysts with a long-axis diameter ≥ 20 mm showed no obvious correlation with the occurrence of pneumothorax.

**Table 3:** The distribution of pulmonary cysts in pneumothorax and non-pneumothorax groups and corresponding P values

	Pneumothorax group [median (quartile)]	Non-pneumothorax group [median (quartile)]	P value
Long-axis diameter of the largest cysts (mm)	46 (28, 54)	28 (12.5, 35.5)	0.028
Short-axis diameter of the largest cysts (mm)	24 (16.8, 36.5)	13 (7, 16.5)	0.004
Volume of the largest cysts (mm^3^)	14,178.5 (4462.3, 35,189.5)	1571 (356, 5438.5)	0.005
Total long-axis diameter of the cysts with long-axis diameter ≥ 20 mm (mm)	149 (71.3, 515.3)	156.5 (26, 403.3)	0.691
Total short-axis diameter of the cysts with long-axis diameter ≥ 20 mm (mm)	77 (24.5, 319)	96 (11.8, 209.8)	0.691
Total volume of the cysts with long-axis diameter ≥ 20 mm (mm^3^)	25,539.62 (4818.8, 109,694.7)	23,126.97 (2221.2, 61,020.9)	0.760
*Right lung*			
Total number of the cysts in the right lung	49 (25, 96.5)	28 (8.5, 56.5)	0.117
Number of the cysts with long-axis diameter ≤ 10 mm in the right lung	36 (20.8, 64.8)	22 (8, 45)	0.171
Number of the cysts with long-axis diameter 10-20 mm in the right lung	9 (2, 16)	2 (0, 11)	0.215
Number of the cysts with long-axis diameter ≥ 20 mm in the right lung	2 (1, 9.8)	0 (0, 4)	0.103
Number of the mediastinal subpleural cysts with long-axis diameter ≤ 10 mm in the right lung	9 (6.5, 12.8)	6 (3, 9.5)	0.154
Number of the mediastinal subpleural cysts with long-axis diameter 10-20 mm in the right lung	3 (1.8, 6)	1 (0, 5)	0.375
Number of the mediastinal subpleural cysts with long-axis diameter ≥ 20 mm in the right lung	1 (0, 6)	0 (0, 1.5)	0.182
Number of the chest wall subpleural cysts with long-axis diameter ≤ 10 mm in the right lung	12.5 (6.5, 29)	9 (1.5, 15.5)	0.177
Number of the chest wall subpleural cysts with long-axis diameter 10-20 mm in the right lung	1.5 (0.8, 4.3)	0 (0, 3)	0.226
Number of the chest wall subpleural cysts with long-axis diameter ≥ 20 mm in the right lung	1 (0, 1.3)	0 (0, 0.5)	0.191
Number of the interlobar subpleural cysts with long-axis diameter ≤ 10 mm in the right lung	6 (3.5, 10.3)	1 (0, 7)	0.223
Number of the interlobar subpleural cysts with long-axis diameter 10-20 mm in the right lung	0 (0, 3.3)	0 (0, 1.5)	0.420
Number of the interlobar subpleural cysts with long-axis diameter ≥ 20 mm in the right lung	1 (0, 2.3)	0 (0, 1)	0.292
*Left lung*			
Total number of the cysts in the left lung	52 (17.8, 85.3)	16 (8.5, 71.5)	0.246
Number of the cysts with long-axis diameter ≤ 10 mm in the left lung	34.5 (14, 59.8)	15 (4, 53.5)	0.307
Number of the cysts with long-axis diameter 10-20 mm in the left lung	11.5 (3.5, 17)	2 (0.5, 12)	0.097
The number of the cysts with long-axis diameter ≥ 20 mm in the left lung	2 (0.8, 8)	1 (0, 7)	0.592
Number of the mediastinal subpleural cysts with long-axis diameter ≤ 10 mm in the left lung	8 (3, 16.3)	4 (2, 15)	0.632
Number of the mediastinal subpleural cysts with long-axis diameter 10-20 mm in the left lung	3.5 (1.5, 5.5)	1 (0, 5)	0.500
Number of the mediastinal subpleural cysts with long-axis diameter ≥ 20 mm in the left lung	1 (0, 2.5)	0 (0, 4.5)	0.823
Number of the chest wall subpleural cysts with long-axis diameter ≤ 10 mm in the left lung	11.5 (4.3, 19.5)	7 (1, 21.5)	0.485
Number of the chest wall subpleural cysts with long-axis diameter 10-20 mm in the left lung	4 (0, 5)	2 (0, 3)	0.145
Number of the chest wall subpleural cysts with long-axis diameter ≥ 20 mm in the left lung	0 (0, 3.5)	0 (0, 1.5)	0.408
Number of the interlobar subpleural cysts with long-axis diameter ≤ 10 mm in the left lung	3.5 (1, 5.5)	0 (0, 4.5)	0.194
Number of the interlobar subpleural cysts with long-axis diameter 10-20 mm in the left lung	1 (0, 2)	0 (0, 1)	0.153
Number of the interlobar subpleural cysts with long-axis diameter ≥ 20 mm in the left lung	0 (0, 0.3)	0 (0, 1)	0.791
*Both lungs*			
Total number of the cysts in both lungs	135 (39, 169.5)	44 (18, 138.5)	0.057
Number of the cysts with long-axis diameter ≤ 10 mm in both lungs	81.5 (32.5, 135)	36 (12.5, 100.5)	0.161
Number of the cysts with long-axis diameter 10-20 mm in both lungs	17 (5.5, 33.5)	5 (0.5, 23)	0.116
Number of the cysts with long-axis diameter ≥ 20 mm in both lungs	5 (2, 19)	1 (0, 11)	0.065
Number of the mediastinal subpleural cysts with long-axis diameter ≤ 10 mm in both lungs	21 (9.8, 27.5)	12 (5, 27.5)	0.324
Number of the mediastinal subpleural cysts with long-axis diameter 10-20 mm in both lungs	7.5 (3.5, 11.5)	2 (0.5, 10)	0.226
Number of the mediastinal subpleural cysts with long-axis diameter ≥ 20 mm in both lungs	1.5 (0.8, 8)	1 (0, 5.5)	0.398
Number of the chest wall subpleural cysts with long-axis diameter ≤ 10 mm in both lungs	26 (13.3, 45.8)	16 (4, 38.5)	0.215
Number of the chest wall subpleural cysts with long-axis diameter 10-20 mm in both lungs	5 (1, 10.3)	2 (0, 5.5)	0.189
Number of the chest wall subpleural cysts with long-axis diameter ≥ 20 mm in both lungs	1.5 (0, 6)	0 (0, 2.5)	0.248
Number of the interlobar subpleural cysts with long-axis diameter ≤ 10 mm in both lungs	9 (5.8, 17.3)	2 (0, 8.5)	0.061
Number of the interlobar subpleural cysts with long-axis diameter 10-20 mm in both lungs	1 (0.8, 5.3)	0 (0, 3)	0.054
Number of the interlobar subpleural cysts with long-axis diameter ≥ 20 mm in both lungs	1 (0, 3.3)	0 (0, 2)	0.234

## Discussion

BHD syndrome is a rare autosomal dominant genetic disease, which is often misdiagnosed as pulmonary bullae. In this study, we included 26 patients with BHD and observed their chest CT and clinical features. The cysts of BHD patients were mainly of round and irregular shape, characterized by subpleural distribution. About 54% of BHD patients developed pneumothorax, and the occurrence of pneumothorax was related to the long-axis diameter, short-axis diameter and volume of the largest cysts (P < 0.05).

The mutation of the folliculin (*FLCN*) gene is closely related to the occurrence of BHD syndrome. BHD syndrome is named after the Canadian doctors Birt, Hogg, and Dubé who reported a family with fibrofolliculomas, trichodiscomas, and acrochordons in 1977. Benign tumors of the skin, various types of renal tumors, and multiple lung cysts are the characteristic manifestations of BHD syndrome. Skin damage mainly manifests as fibrofolliculomas, which may be accompanied by trichodiscomas and acrochordons. Mixed oncocytoma or chromophobe cell tumor is a typical renal manifestation [3]. Epidemiological data have shown that the incidence of tumors in the skin and lungs is higher than that of renal tumors [4]. Skin lesions and *FLCN* gene mutations are classified as the main diagnostic criteria of BHD syndrome [5]. There are ethnic differences in the clinical manifestations of BHD syndrome. Previous studies have shown that > 80% of BHD syndrome cases in Europe and the United States have specific skin lesions, while the incidence of skin lesions and renal tumors in Asians is low [6–8]. There were no typical skin and kidney lesions in the cases included in our study.

A majority of patients with BHD syndrome have pulmonary cysts [2, 9–11]. In our study, lung cysts were found in all the 23 (100%) patients’ chest CT images. The cysts in the lungs of all patients were bilateral and multiple, with different sizes. The larger cysts were often irregular, while the smaller cysts were mostly round or oval. Some of the larger cysts near the mediastinal spine showed plastic changes along the mediastinum and spine, which may have been caused by high pressure in the capsule, with limited growth space. In the current study, 76.3% of the cysts were distributed under the pleura of both lungs, and 93.6% of the cysts with a long-axis diameter > 20 mm were distributed under the pleura, especially near the mediastinum (48.4%). Furuya and Koga et al. [12, 13]studied the unruptured pulmonary cysts associated with BHD syndrome and found that the cyst wall expanded toward the visceral pleura and was partially embedded in parenchyma, interlobular septum, and bronchovascular bundles. Additionally, enlarged cysts were segmented by the alveolar wall and deeply embedded in the interlobular septum. These complex structures were usually accompanied by varying degrees of chronic inflammatory cell infiltration, suggesting possible inflammation-induced development of cysts. Cystic alveoli and fusion of the epithelium of the cyst to the mesenchyme are indicators for BHD-associated lung lesions [14]. BHD syndrome-associated cysts are originally located close to the interlobular septum or subpleura [14], which could explain why 76.3% of the cysts on chest CT images in our study were under the pleura of both lungs. Our study showed that in addition to subpleural cysts of the mediastinum and chest wall, multiple cysts were also observed in the bilateral interlobar pleura. This finding is consistent with the above-mentioned pathological results. Despite the existence of multiple cysts in the lungs, the patients’ pulmonary function remains unaffected [15]. In our study, a BHD patient with normal pulmonary function had experienced pneumothorax for four times and received surgical treatment.

Lung-associated symptoms are usually the first to appear in patients with BHD syndrome. Pneumothorax may be the first and only symptom that causes most patients with BHD syndrome to visit the hospital. Though classified as minor criteria for the diagnosis of BHD syndrome, pulmonary CT findings are the main indication for BHD syndrome that can prompt patients to undergo further examinations to determine the diagnosis. Many studies have shown that the prevalence of spontaneous pneumothorax in patients with BHD syndrome reaches > 60% [6, 8, 16, 17]. Additionally, the risk of pneumothorax in patients with BHD syndrome is 50 times that of family members without BHD syndrome [4]. In our study, 54% of the patients with BHD syndrome developed pneumothorax, of whom 43% had it twice or more times. We deemed that the high incidence of pneumothorax in patients with BHD syndrome, especially recurrent pneumothorax, was related to their lung cysts. Therefore, we carefully examined the patients’ chest CT images and classified them according to the distribution and size of the lesions. We found that the long-axis diameter, short-axis diameter, and volume of the largest cyst were correlated with the occurrence of pneumothorax, especially the short-axis diameter and volume of the largest cyst. This finding may be related to the shape and tension of the capsule, because the short-axis diameter can better reflect tension of the cyst. For capsules of the same volume, the larger the short-axis diameter is, the closer the cyst is to the spheroid and the greater the tension is. As the tension increases, the risk of bursa rupture is higher under the same condition.

In our study, the cysts with long-axis diameter ≤ 10 mm were the most common in both lungs. However, we thought that cysts with long-axis diameter ≥ 20 mm, especially the subpleural ones, were more characteristic, which could help to diagnose BHD. Toro et al.[2] found that the exon position of a BHD mutation was related to the number of lung cysts, as well as the size and volume of the largest cyst. This finding indicates that an *FLCN* mutation may play an important role in the occurrence of pneumothorax. Pathologically abnormal epithelial/mesenchymal interactions may weaken the extracellular matrix of the visceral pleura, leading to pneumothorax [14].

The occurrence of pneumothorax in patients with BHD syndrome is not only related to the patient’s lung lesions, but also related to the external environment and various inducements. Johannesma et al. [9] found that flying or diving-associated air pressure changes may increase the risk of pneumothorax for patients with BHD syndrome, and the risk of pneumothorax is higher during flight. It is suggested that BHD patients with presence of any clinical symptoms (such as shortness of breath or chest pain) during flight or shortly after air travel should receive a chest X-ray or CT examination to exclude pneumothorax. In our study, one patient developed pneumothorax after returning from a trip to Yunnan plateau region by plane and another patient developed pneumothorax after dumbbell lifting. We speculate that the occurrence of pneumothorax in these patients was related to the change in atmospheric pressure and thoracic pressure. For patients with recurrent pneumothorax, pleural closure/covering technology [18] or the combined intervention with mechanical and chemical pleurodesis [19] can effectively treat pneumothorax and reduce recurrence.

In our study, 4 patients developed lung tumors (including small cell lung cancer, adenocarcinoma, and alveolar cell tumor). There is limited information currently available about the incidence of lung cancer in patients with BHD syndrome. Notably, lung adenocarcinoma has been reported in heterozygous *FLCN* knockout mice [20]. Whether *FLCN* gene mutations increase the risk of tumorigenesis in BHD patients requires further investigation.

Chest CT findings of BHD syndrome are characterized by bilateral, multiple, irregular shaped cysts of various sizes with subpleural predominance. Our quantitative analysis of the number, size, and distribution of lung cysts showed that there was a correlation between lung cysts and the occurrence of pneumothorax. Clinicians (especially emergency department physicians and surgeons) need to obtain more information about the disease history and family history when treating patients with pneumothorax. For patients with suspected BHD syndrome, a chest CT examination should be used rather than X-ray for more comprehensive assessment of lung lesions, and gene testing and a visit to the dermatology clinic are recommended. Following early detection of this syndrome, the patients and their families should be followed up, given personalized advice on diving and other related exercises, and pay close attention to pneumothorax and renal tumors that may affect prognosis and quality of life.

There are some limitations of our study. First, our study was a small sample retrospective study, which may lead to potential bias, uncertainty and generality in our statistical results. Second, some patients had a history of pneumothorax, which may affect the characteristic morphology, distribution, and number of pulmonary cysts. Therefore, we will enroll more patients in prospective design studies in the future to better observe the characteristics of pulmonary cysts and explore their relationship with pneumothorax in patients with BHD.

## Conclusion

BHD patients have specific pulmonary imaging findings, and the occurrence of pneumothorax in BHD patients is closely related to their pulmonary cystic lesions.

## Data Availability

The datasets used and/or analyzed during the current study are available from the corresponding author on reasonable request.

## Abbreviations

BHD: Birt–Hogg–Dubé
CT: Computed tomography
FLCN: Folliculin
